# Proteins involved in embryo-maternal interaction around the signalling of maternal recognition of pregnancy in the horse

**DOI:** 10.1038/s41598-018-23537-6

**Published:** 2018-03-27

**Authors:** Katrien Smits, Sander Willems, Katleen Van Steendam, Margot Van De Velde, Valérie De Lange, Cyrillus Ververs, Kim Roels, Jan Govaere, Filip Van Nieuwerburgh, Luc Peelman, Dieter Deforce, Ann Van Soom

**Affiliations:** 1 Department of Reproduction, Obstetrics and Herd Health, Faculty of Veterinary Medicine, Ghent University, Merelbeke, Belgium; 2 Laboratory of Pharmaceutical Biotechnology, Faculty of Pharmaceutical Sciences, Ghent University, Gent, Belgium; 3 Laboratory of Animal Genetics, Faculty of Veterinary Medicine, Ghent University, Merelbeke, Belgium

## Abstract

During maternal recognition of pregnancy (MRP), a conceptus-derived signal leads to the persistence of the corpus luteum and the maintenance of gestation. In the horse, the nature of this signal remains to be elucidated. Several studies have focused on the changes in gene expression during MRP, but little information exists at the protein level. The aim of this study was to identify the proteins at the embryo-maternal interface around signalling of MRP in the horse (day 13) by means of mass spectrometry. A distinct influence of pregnancy was established, with 119 proteins differentially expressed in the uterine fluid of pregnant mares compared to cyclic mares and with upregulation of several inhibitors of the prostaglandin synthesis during pregnancy. By creating an overview of the proteins at the embryo-maternal interface in the horse, this study provides a solid foundation for further targeted studies of proteins potentially involved in embryo-maternal interactions, MRP and pregnancy loss in the horse.

## Introduction

Maternal recognition of pregnancy (MRP) covers the series of events leading to the persistence of the corpus luteum and a receptive uterine environment to support the maintenance of gestation^1^. In the cycling mare, pulsatile release of prostaglandin F2α (PGF2α) causes luteolysis, resulting in a decline in progesterone. This mechanism is inhibited during pregnancy by the presence of the conceptus^2^. In pigs, the conceptus derived signal which initiates MRP has been identified a long time ago as oestrogen^3^ and in ruminants as interferon tau^4,5^. However, the nature of this signal remains to be elucidated in the horse despite several decades of elaborate research on this topic^6,7^. Initial studies have focused on the identity of specific candidate signalling molecules and while the equine embryo produces substantial quantities of oestrogen as well as prostaglandins (PG) and limited amounts of interferons, no convincing evidence exists for their signalling role in MRP^7^. Potential embryonic signal targets involved in the luteostatic mechanism in the horse are prostaglandin-endoperoxide synthase 2 (PTGS2), an enzyme in the biosynthesis of PGF2α, and oxytocin, which stimulates endometrial PGF2α secretion through a positive feedback loop^8^. Both *PTGS2* and oxytocin receptor expression (OXTR) are repressed during early pregnancy compared to cycling mares, with downregulation of *PTGS2* at the RNA level and of OXTR at the protein level^9–13^.

During the last years, the topic of MRP in the horse has been broadened to all pathways involved in embryo-maternal communication around the timing of MRP. Signalling of MRP is a continuum of events, estimated to occur between days 12 and 14. Recipient mares can still get pregnant when an embryo is transferred to their uterus at day 12, but not at day 14 after ovulation^14^, while repression of *PTGS2* occurs by day 13 of pregnancy^11^. By day 16, clear differences between pregnant and cyclic horses are observed. Transcriptomics of the equine endometrium and equine conceptuses have substantially contributed to the knowledge on pathways affected around the timing of MRP in the horse^7,15–19^. Technological advantages, including sequencing, favoured development of genomics and transcriptomics compared to proteomics^20^. However, mRNA abundances can only explain 40% of the variation in protein levels and the actual protein profile is influenced by post-transcriptional regulation mechanisms^21^. This appeals for complementing transcriptomics knowledge on MRP with quantitative proteomics. This can now be achieved through mass spectrometry (MS). Recent improvements in MS technologies, including data-independent-acquisition, allow reproducible label-free quantification of proteins in complex biological samples^22^.

Mass spectrometry of the embryo-maternal interface around MRP has been performed in several farm animals including pigs^23,24^, sheep^25^ and cattle^26–28^. In the horse, specific molecules with a potential role in MRP have been targeted by immunohistochemistry^13,29–32^ and global screening of uterine proteins has been performed in the context of endometritis^33^. However, the effect of pregnancy on the uterine secretome has not been assessed by means of high-throughput proteomics in the horse up to now. In a recent study, equine blastocysts were collected by uterine lavage on day 8 and an MS analysis was performed of the proteins secreted during culture of these embryos for 24 h and 48 h and of proteins present in the blastocoel fluid and the embryo capsule^34^. The authors detected prostaglandin F2 receptor inhibitor (PTGFRN) and a progesterone potentiating protein, FK506 binding protein 4 (FKBP4), in the blastocoel fluid, but it remained to be determined whether these proteins were actively secreted into the uterine lumen.

The aim of this study was to gain new insights into the embryo-maternal communication around the signalling of MRP in the horse. Since signalling of MRP is estimated to occur between Day 12 and Day 14, sampling was performed at Day 13 (±0.5 day). We hypothesize that high-throughput proteomics can provide complementary information to the transcriptomic reports. To this end, proteomics was performed by high definition data independent mass spectrometry (HDMS^E^) with ion mobility drift time-specific collision-energy^35^. In this way, proteins were identified and quantified in uterine fluid of pregnant and cyclic mares as well in the yolk sac fluid of the pregnant mares.

## Results

### Sampling

Only reproductively sound mares with negative bacteriology and cytology of the uterine fluid were used for the sampling. In two cycles, namely one pregnant (P) and one control cyclic (C) cycle, a double ovulation occurred. Response to hCG resulted in ovulation 24–36 h after administration. In four cycles, ovulation only occurred 3 days after hCG; once in a P cycle, where artificial insemination (AI) was performed at the same time and in this case, the mare was inseminated a second time 48 h after the first time and she ovulated the day after. In all other P cycles, ovulation occurred within 48 h after AI. In one mare, a line of fluid was noticed by ultrasound of the uterus 1 day after AI and she was treated by intramuscular administration of oxytocin.

### Identification of proteins

The average protein concentration was similar in the uterine fluid (UF) of P (9.2 g/mL) and C (9.8 g/mL) mares, while the average protein concentration in the yolk sac (YS) was only 78 µg/mL.

For the first time, an overview was created of the proteins present in the UF and the YS at day 13 after ovulation in the horse. In the UF samples, a total of 10489 peptides were identified, accounting for 41% of all peptide like ions. Protein identification resulted in 1153 identifiable proteins (Supplementary file 1). After filtering and normalization, a total of 707 normalized proteins with at least two unique peptides were assessed for differential expression.

Differential expression of proteins was assessed for P versus C mares and pregnancy was associated with upregulation of 62 proteins (Table 1) and downregulation of 57 proteins (Table 2). For all proteins in this comparison, the log fold change, the adjusted p-value and the number of peptides are listed in Supplementary file 3.

**Table 1:** Upregulated proteins in the uterine fluid of pregnant versus cyclic mares on day 13 after ovulation.

Protein Symbol	Log FC	Adj. p-value	Gene Symbol	Gene Description
F7BAA0	2,27	0,03617	GSTO1*	glutathione S-transferase omega 1
F6Z0A9	2,02	0,01980	RAC1*	ras-related C3 botulinum toxin substrate 1 (rho family, small GTP binding protein Rac1)
F6VVU1; F6YMX5	1,96	0,02370	MOB1A*	MOB kinase activator 1A
F6Y2H3; F6Y2V7	1,89	0,02907	PEPD*	peptidase D
F7CCF5	1,81	0,01425	LXN*	latexin
F7DIB3	1,80	0,03253	SEC14L3*	SEC14 like lipid binding 3
F6YAZ9; F7BYZ9	1,79	0,01633	MYL12A*	myosin light chain 12A
F6RH25	1,55	0,01371	DCPS*	decapping enzyme, scavenger
F6XV30	1,55	0,01980	TBCA*	tubulin folding cofactor A
Q3S4D6	1,53	0,01915	GM2A	GM2 ganglioside activator
F6XKI9	1,40	0,01142	DNTTIP2*	deoxynucleotidyltransferase terminal interacting protein 2
F6RTH0	1,39	0,01378	TXNDC17*	thioredoxin domain containing 17
F7CBN0	1,35	0,00336	AKR1A1	aldo-keto reductase family 1 member A1
F6PWC8	1,25	0,00112	PTGR1*	prostaglandin reductase 1
F6XSN2	1,24	0,00106	CCT7*	chaperonin containing TCP1 subunit 7
F6XZQ1	1,24	0,00026	CAPS*	calcyphosine
F7BAR2	1,23	0,02275	TPMT	Thiopurine S-methyltransferase
F6W8C8	1,20	0,00004	SERPINB6*	serpin family B member 6
F6RGN2	1,19	0,04597	FABP5*	fatty acid binding protein 5
F6RMM1	1,17	0,00336	SH3BGRL	SH3 domain binding glutamate rich protein like
F7CBR0; F7DZD2	1,11	0,00336	LOC100050322	Glutathione S-transferase
F7BHV8	1,11	0,00626	TUBB4A*	tubulin beta 4A class IVa
Q8HZM6; F7A0T0	1,09	0,00106	ANXA1	Annexin A1
F6XA04	1,06	0,00001	YWHAE*	tyrosine 3-monooxygenase/tryptophan 5-monooxygenase activation protein epsilon
F6XTY8	1,05	0,02235	Unassigned	unassigned
F7D3E3	1,03	0,00004	CMPK1*	cytidine/uridine monophosphate kinase 1
F6W683	0,99	0,03617	GMDS*	GDP-mannose 4,6-dehydratase
F7DB59	0,99	0,00106	PAFAH1B3*	platelet activating factor acetylhydrolase 1b catalytic subunit 3
F6SQ49	0,97	0,00112	SMS*	spermine synthase
F6RL46	0,96	0,01211	PGLS*	6-phosphogluconolactonase
F6W9B1	0,93	0,01371	ST13	suppression of tumorigenicity 13 (colon carcinoma) (Hsp70 interacting protein)
F7E0H3	0,91	0,01211	TUBB*	tubulin beta class I
F6R8T8	0,90	0,01473	ACY1	aminoacylase 1
F7D9J2	0,90	0,00053	TKT*	transketolase
F6W039	0,85	0,00024	ARHGDIA*	Rho GDP dissociation inhibitor alpha
F6ZHQ5	0,83	0,00336	CLIC1	chloride intracellular channel 1
F7D1R1	0,82	0,01371	PGK1	Phosphoglycerate kinase 1
F6TZS9	0,77	0,01633	TPI1	triosephosphate isomerase 1
F7CIX6	0,76	0,00591	ENO2*	enolase 2
F6W3T1	0,73	0,00106	LDHA	lactate dehydrogenase A
F7C5G3	0,73	0,01378	PSMD11*	proteasome 26S subunit, non-ATPase 11
F6ZE54	0,72	0,04589	GPI	glucose-6-phosphate isomerase
F7BWW6	0,71	0,02824	VCP*	valosin containing protein
F7CZS6	0,71	0,01052	MDH1	malate dehydrogenase 1
F6PJY2	0,71	0,04256	LZTFL1*	leucine zipper transcription factor like 1
F7DMY1	0,70	0,04029	CBFB*	core-binding factor beta subunit
F6UJ33	0,69	0,02943	PFN1	profilin
F6VSN2	0,69	0,00056	GSTP1*	glutathione S-transferase pi 1
F7DXG8	0,69	0,01915	CFL1*	cofilin 1
F7APS1; F6ZWS7	0,68	0,00106	CSTB; LOC100050835*	Cystatin B
F7BE95; F6U2P8	0,67	0,03059	UBE2V1*	ubiquitin conjugating enzyme E2 V1
F7ALV0	0,67	0,00336	TXN	Thioredoxin
F7BPT4	0,61	0,00336	EZR*	Ezrin
F6S5E7	0,59	0,01211	TARS*	threonyl-tRNA synthetase
F6QXW2	0,58	0,01378	PEBP1*	phosphatidylethanolamine binding protein 1
F6XLG0; F7DY67	0,57	0,04597	PNP; LOC100058767	Purine nucleoside phosphorylase
F7DZV9	0,57	0,00961	YWHAB*	tyrosine 3-monooxygenase/tryptophan 5-monooxygenase activation protein beta
F7CI32; F7ASU6; F7DKR3	0,51	0,03433	SELENBP1*	selenium binding protein 1
F7B5P1	0,49	0,03253	CNDP2*	CNDP dipeptidase 2 (metallopeptidase M20 family)
F6YZ13	0,48	0,03604	S100A13*	S100 calcium binding protein A13
F6ZEV8	0,46	0,01618	DBI*	diazepam binding inhibitor, acyl-CoA binding protein
F6X6A6; F6XKX6; F6Z5Z4	0,45	0,00423	LOC100052020; LOC100054282	Peptidyl-prolyl cis-trans isomerase

**Table 2:** Downregulated proteins in the uterine fluid of pregnant versus cyclic mares on day 13 after ovulation.

Protein ID	Log FC	adj. P-Value	Gene Symbol	Gene Description
F6USV6	−0,44	0,04220	NOL11*	nucleolar protein 11
F7BF31	−0,52	0,01207	SPI2*	alpha-1-antitrypsin
F6WZW6	−0,57	0,01980	PSMA1	proteasome subunit alpha 1
F6YLA3	−0,62	0,00626	TXNRD1*	thioredoxin reductase 1
F6YVT0	−0,71	0,01004	RASGRP4*	RAS guanyl releasing protein 4
F6ZFH9	−0,72	0,02902	YWHAG*	tyrosine 3-monooxygenase/tryptophan 5-monooxygenase activation protein gamma
F7AED2	−0,72	0,01528	LOC100050100*	alpha-1-acid glycoprotein 2-like
F7CZW9	−0,73	0,02003	SERPING1*	serpin family G member 1
F6T7X3	−0,75	0,00626	LOC100065767	membrane primary amine oxidase
F6RRV1	−0,76	0,00368	FETUB*	fetuin B
F7BKK5	−0,76	0,04705	GSTM3*	glutathione S-transferase mu 3
F6PQ46	−0,78	0,01052	CP*	ceruloplasmin
F6R942; F6RI47	−0,79	0,00336	A2M*	Alpha-2-macroglobulin
F6SJ41	−0,82	0,03640	PFN2	profilin 2
F6XWM5	−0,82	0,00041	HP*	haptoglobin
F6RMD0	−0,87	0,00516	CFB*	complement factor B
F6ZD04	−0,89	0,01443	PYGB	glycogen phosphorylase B
P69905	−0,89	0,01010	HBA1	hemoglobin subunit alpha 1
F7AJP3	−0,90	0,02643	CHI3L1*	chitinase 3 like 1
F6VTZ7	−0,91	0,00072	CFAP58*	cilia and flagella associated protein 58
F6RDD3; F6VE37	−0,91	0,00072	HBB	hemoglobin subunit beta
F7BFJ1	−0,92	0,01242	F2	coagulation factor II, thrombin
F6RZ27	−0,93	0,00336	APOA4*	apolipoprotein A4
F6WMT7; F7C7Y1	−0,93	0,00053	KRT71; KRT73*	keratin 71; keratin 73
F6QS41; F7BQS9	−0,95	0,00336	MROH2A*	maestro heat like repeat family member 2A
F6Z2L5	−0,96	0,00005	APOA1*	apolipoprotein A1
F6XM13	−0,98	0,01371	APOD*	apolipoprotein D
F6XLB1	−0,99	0,00056	LTF	Lactotransferrin
F6XRU1; F6YAV2	−1,01	0,00217	SERPINB11*	serpin family B member 11
F7DTV1	−1,04	0,01115	PON1*	paraoxonase 1
F6SGV0	−1,07	0,00338	TTF2*	transcription termination factor 2
Q29482	−1,07	0,01298	CLU	clusterin
F6SRP7	−1,10	0,01010	CAP1	adenylate cyclase associated protein 1
F6QX36	−1,13	0,00119	ITIH1*	inter-alpha-trypsin inhibitor heavy chain 1
F6TJX5	−1,16	0,03059	TPP1*	tripeptidyl peptidase 1
F7BZ41	−1,19	0,02235	CTSL*	cathepsin L
F7BNQ2	−1,20	0,00217	C4BPA*	complement component 4 binding protein alpha
F7AMJ7	−1,20	0,02095	STK38*	serine/threonine kinase 38
F6RM73	−1,22	0,00366	APOA2	Apolipoprotein A-II
F6YNT8	−1,27	0,02370	PEBP4*	phosphatidylethanolamine binding protein 4
F6VUW2	−1,30	0,00626	CTSS*	cathepsin S
F6TE92	−1,33	0,02043	AGL	amylo-alpha-1, 6-glucosidase, 4-alpha-glucanotransferase
F7C0E6	−1,36	0,00259	PLS1*	plastin 1
F6X5J6	−1,36	0,02043	ADSL	adenylosuccinate lyase
F7BCH1	−1,37	0,00000	INHBA	Inhibin beta A chain
F6PUX2	−1,41	0,00026	MSN*	moesin
F7DXH4	−1,44	0,00106	VIPAS39*	VPS33B interacting protein, apical-basolateral polarity regulator, spe-39 homolog
P01008	−1,54	0,00178	SERPINC1	serpin family C member 1
F7CWC8	−1,57	0,00119	unassigned	Amine oxidase [flavin-containing]
F7CWT0	−1,58	0,00056	P19*	P19 lipocalin
F6WRK2	−1,59	0,00199	MANBA*	mannosidase beta
F7BLE3	−1,69	0,01851	unassigned	unassigned
F6QYS3	−1,75	0,01765	ECM1*	extracellular matrix protein 1
F6R8P9; F6RM27	−2,13	0,00112	TTLL7*	tubulin tyrosine ligase like 7
F6SJN4	−2,22	0,00556	UBOX5*	U-box domain containing 5
F7CHR8	−2,23	0,01530	CCDC36*	coiled-coil domain containing 36
F6VST0; F6W6H2	−3,34	0,00004	NEFL*	neurofilament, light polypeptide

In the YS samples, a total of 6500 peptide ions were identified, representing 51% of all peptide like ions and resulting in 903 identifiable proteins (Supplementary file 2). For the YS proteins, the primary goal was identification, rather than quantification, as different nature of the fluids impedes assessment of differential expression of proteins in YS versus UF.

### Gene Ontology enrichment and pathway analysis

Categorization in the Gene Ontology (GO) terms ‘molecular function’, ‘biological process’ and ‘cellular component’ is provided for all quantified proteins in the comparison of P versus C in Supplementary file 3.

**Figure 1:**
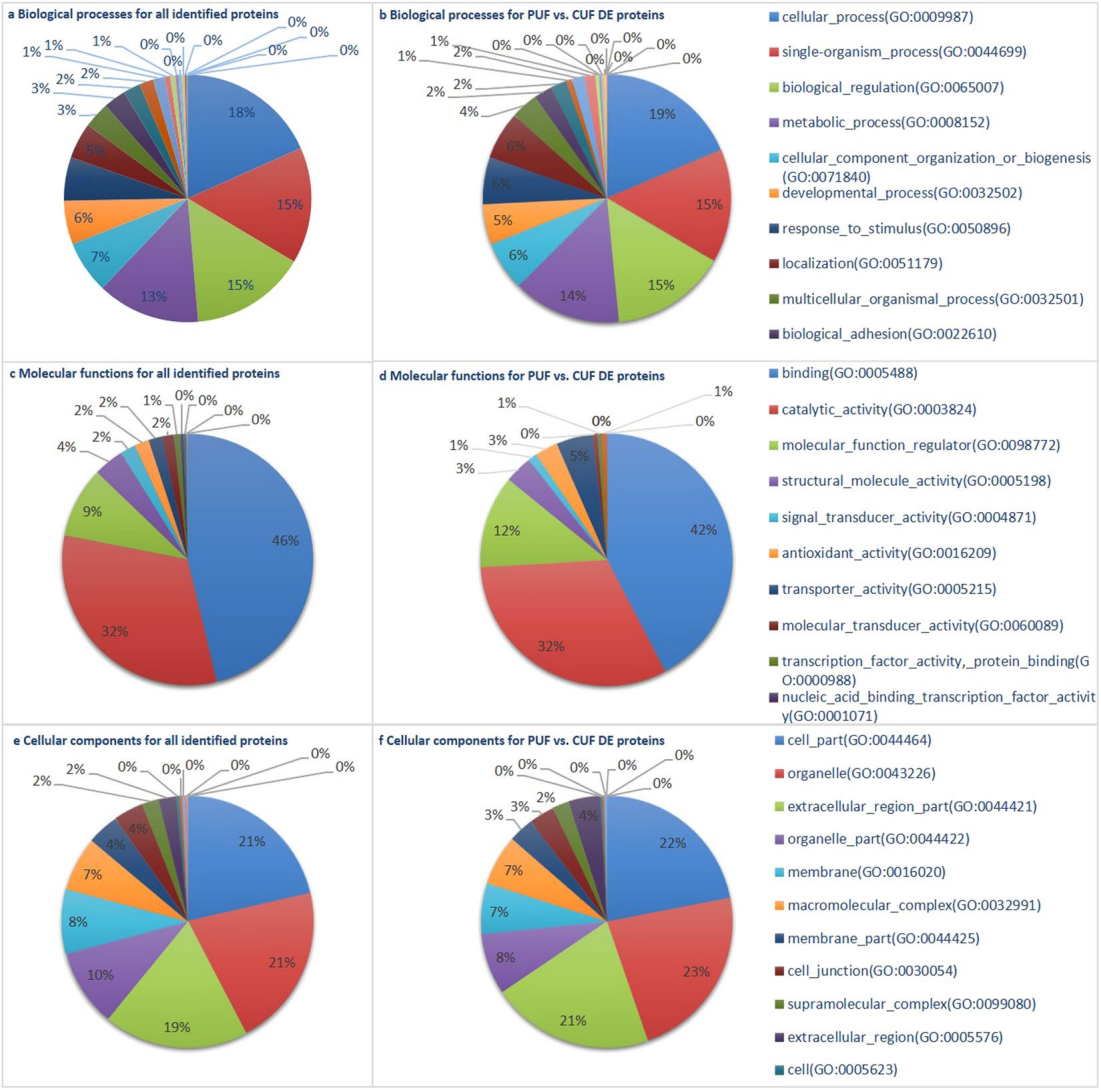
Categorization in Gene Ontology terms of all identified proteins in the uterine fluid (UF) and of differentially expressed (DE) proteins in the uterine fluid of pregnant (P) versus cyclic (C) mares. The main GO biological processes (**a**,**b**), molecular functions (**c**,**d**) and structural components (**e**,**f**) are represented for all quantified proteins in the equine uterine fluid (**a**,**c**,**e**) as well as for proteins found to be differentially expressed in the uterine fluid of pregnant versus cyclic mares (**b**,**d**,**f**).

Figure 1 summarizes the GO categories in which the differentially expressed proteins are involved. The main category to which most proteins are assigned is ‘cellular process (GO:0009987)’ for the biological processes and ‘binding (GO:0005488)’ for the molecular functions. This coincides with the results in porcine uterine fluid^24^, but these are also the major categories when all proteins are taken into account. Overall, the differences in categorization between the groups are small.

Gene Ontology (GO) enrichment revealed no statistical overrepresentation when a Bonferroni correction for multiple testing was used (FDR < 0.05). No up- or downregulated KEGG pathways were detected either at a Benjamini-Hochberg corrected p-value of 0.05.

### Embryo-maternal interaction

**Figure 2:**
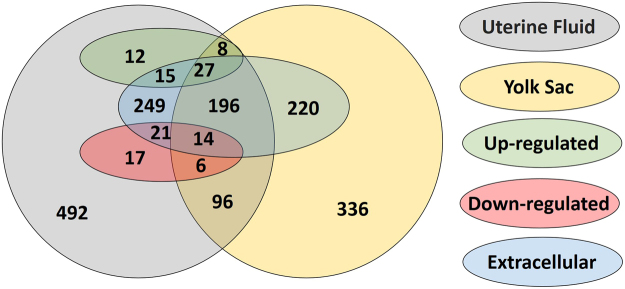
Proteins identified in the uterine fluid and the yolk sac fluid. The number of common proteins as well as the number of proteins specific for the uterine fluid or the yolk sac fluid are displayed. The proteins which were found to be upregulated or downregulated in the uterine fluid of pregnant mares compared to cyclic mares are depicted separately. Proteins categorized in the extracellular space are also indicated.

Comparison of the proteins identified in the UF of the P mares and in the YS of the corresponding embryo revealed 347 common proteins, 806 proteins which were only detected in the UF and 556 proteins which were only found in the YS. Figure 2 represents an overview of these UF specific proteins, YS specific proteins and common proteins, with specific display of the proteins up- and downregulated during pregnancy and of the proteins categorized in the extracellular space.

**Figure 3:**
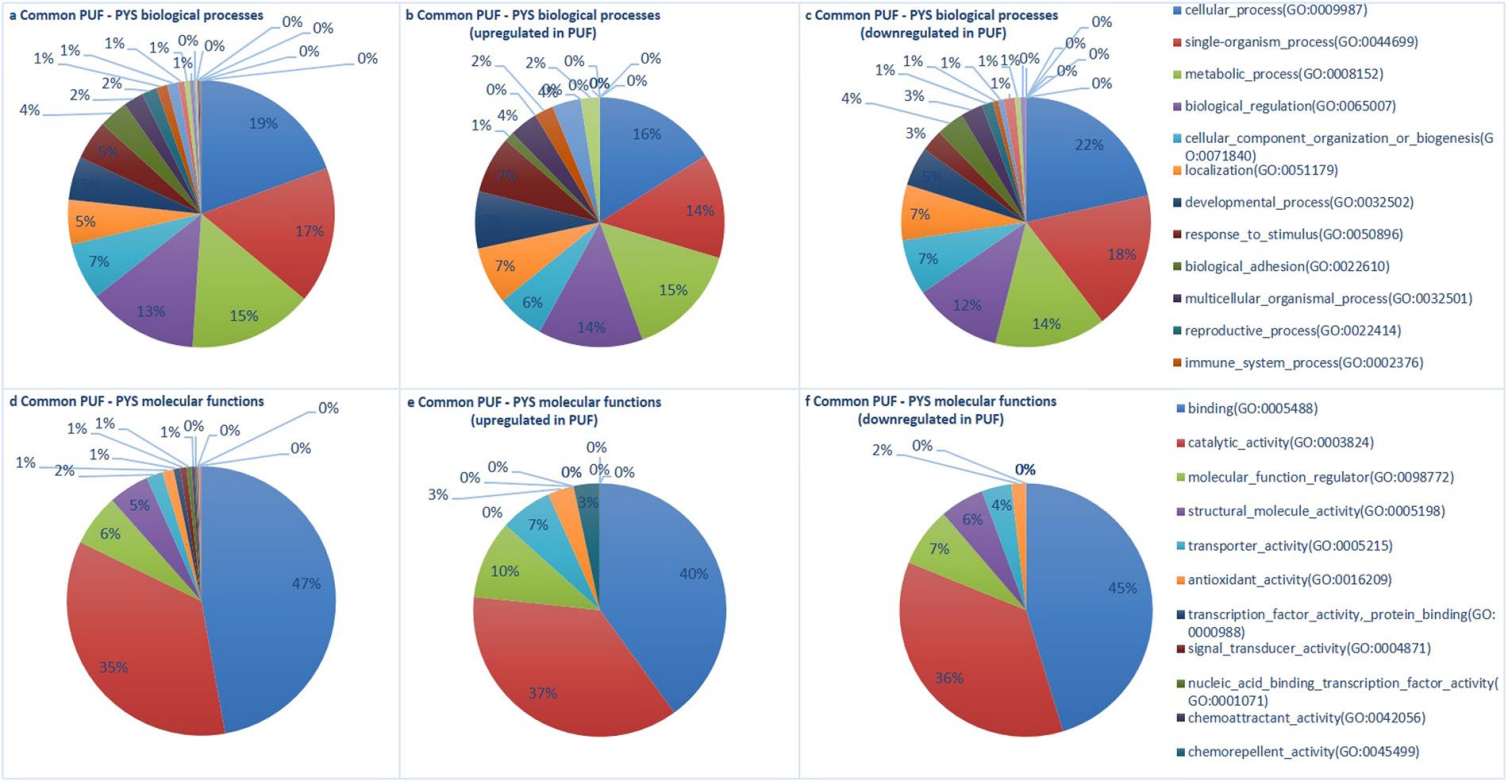
Categorization in Gene Ontology terms of common proteins in the uterine fluid of pregnant mares (PUF) and the yolk sac fluid (PYS) of the corresponding embryos. The main GO biological processes (**a**–**c**) and molecular functions (**d**–**f**) are represented for all common proteins in equine uterine fluid and yolk sac fluid (**a**,**d**), as well as for the subset of common proteins which were found to be upregulated (**b**,**e**) or downregulated (**c**,**f**) in the uterine fluid of pregnant mares compared to cyclic mares.

A list of the 347 common proteins is provided in Supplementary file 4, including the functions in which these proteins are involved. Figure 3 summarizes the GOs in which these common proteins were involved. Similar to the results for the UF in Fig. 1, the main GO categories in which the common proteins are involved are also ‘cellular process (GO:0009987)’ and ‘binding (GO:0005488)’ and differences in categorization are small. Common proteins in YS and UF which were also upregulated in P versus C, showed a higher representation in the biological processes ‘developmental process (GO:0032502)’ and ‘response to stimulus (GO:0050896)’. Molecular functions in which these proteins were more involved are ‘transporter activity (GO:0005215)’ and ‘transcription factor activity - protein binding (GO:0000988)’, while the common proteins which were downregulated in P versus C were rather represented in ‘structural molecule activity (GO:0005198)’.

**Figure 4:**
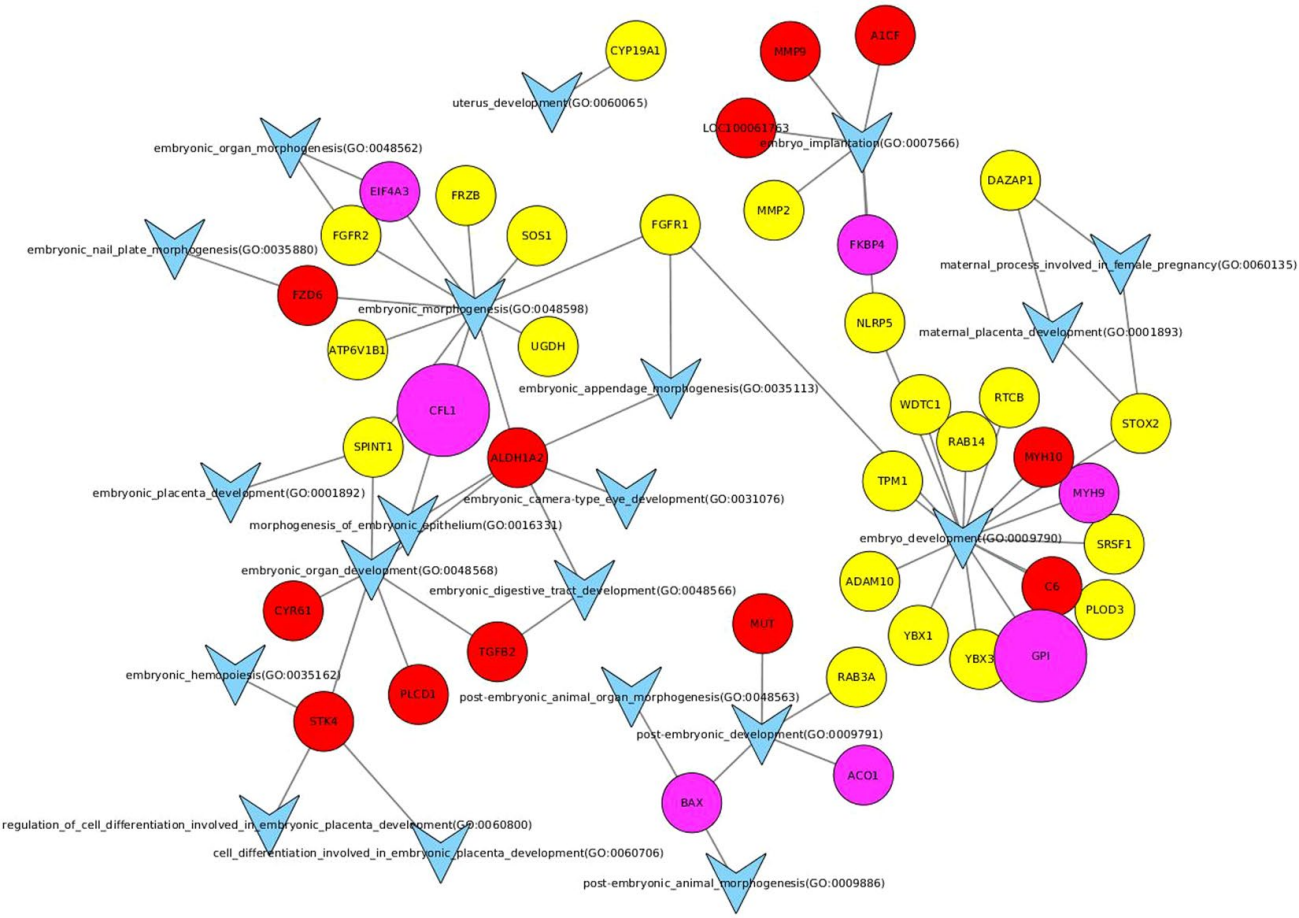
Involvement of proteins found in the yolk sac fluid and the uterine fluid of pregnant mares in GO terms and pathways representing embryo-maternal interaction. All GO terms and pathways that include ‘embryo’, ‘maternal’ or ‘uterus’ in their description were selected, together with all identified proteins in either the yolk sac or uterine fluid of pregnant horses belonging to these GO terms or pathways. These GO terms, pathways and proteins were then visualized using Cytoscape 3.3.0. Proteins found only in the uterine fluid are represented as red circles, proteins found only in the yolk sac as yellow circles and proteins found in both as purple circles. Proteins significantly (FDR corrected p-value < 0.05) up- or downregulated in the uterine fluid of pregnant mares are respectively larger and smaller circles (size not scaled with magnitude of up- or downregulation). GO terms and pathways are represented as a blue ‘V’, with lines indicating whether a GO term or pathway is associated with a protein.

**Figure 5:**
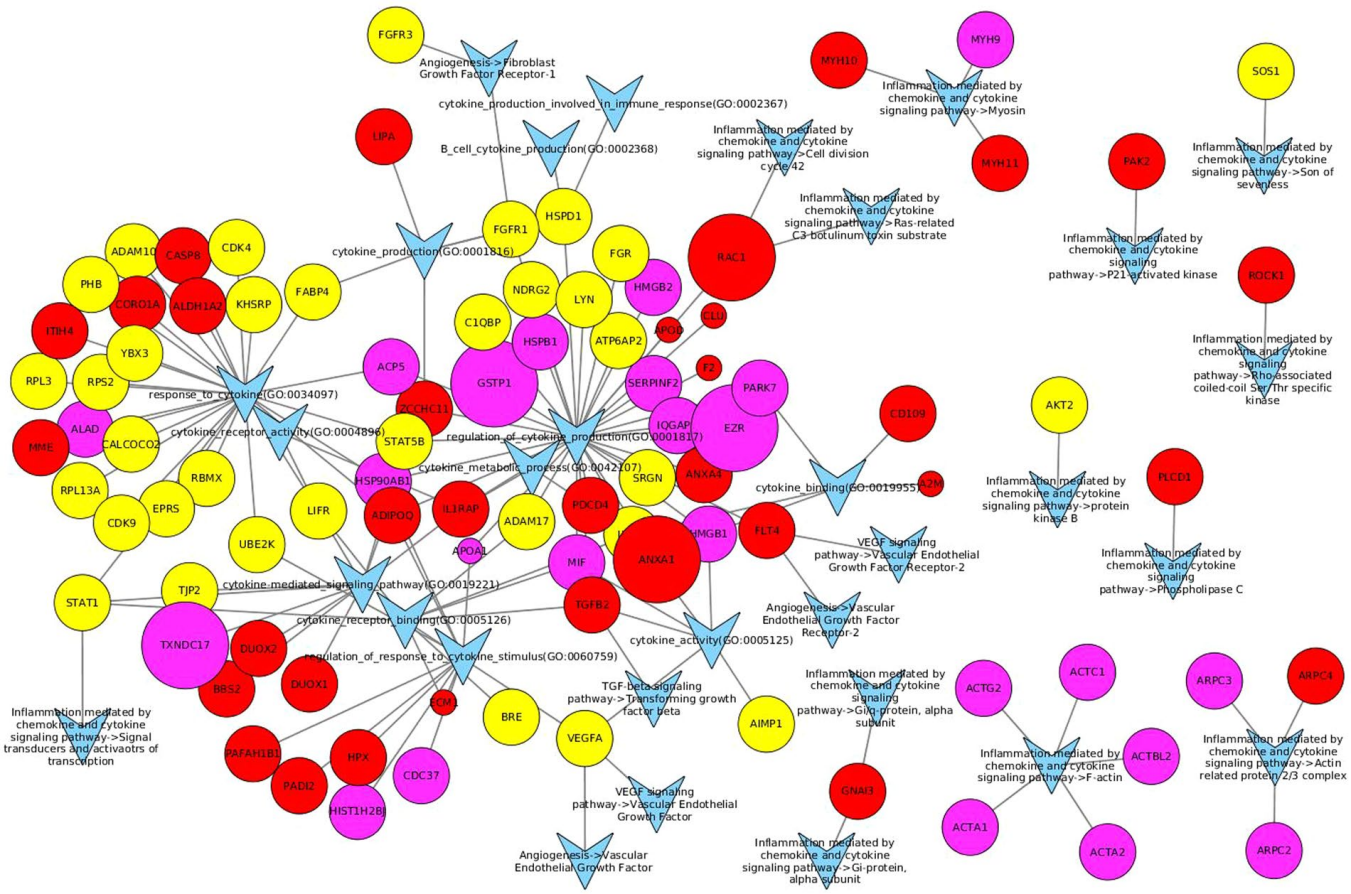
Involvement of proteins found in the yolk sac fluid and the uterine fluid of pregnant mares in GO terms and pathways representing embryo-maternal interaction. All GO terms and pathways that include ‘cytokine’ or ‘growth factor’ in their description were selected, together with all identified proteins in either the yolk sac or uterine fluid belonging to these GO terms or pathways. These GO terms, pathways and proteins were then visualized using Cytoscape 3.3.0. Proteins found only in the uterine fluid are represented as red circles, proteins found only in the yolk sac as yellow circles and proteins found in both as purple circles. Proteins significantly (FDR corrected p-value < 0.05) up- or downregulated in the uterine fluid of pregnant mares are respectively larger and smaller circles (size not scaled with magnitude of up- or downregulation). GO terms and pathways are represented as a blue ‘V’, with lines indicating whether a GO term or pathway is associated with a protein.

The embryo-maternal interaction was further visualized by Cytoscape 3.3.0 in Fig. 4. The most prominent GO terms in this network are ‘embryo development’ (GO:0009790) and ‘embryo morphogenesis’ (GO:0048598), with a main contribution of proteins originating from the yolk sac, and embryo implantation (GO:0007566) with the involvement of both uterine and embryonic proteins. In Fig. 5, the contribution of growth factors and cytokines in equine embryo-maternal signalling is visualized. The most extensive networks with various proteins found in the yolk sac fluid and/or the uterine fluid of pregnant mares are involved in ‘regulation of cytokine production’ (GO:0001817), ‘response to cytokine’ (GO:0034097) and the downstream GO’s ‘cytokine receptor binding‘ (GO:0005126), ‘cytokine mediated signalling pathway’ (GO:0019221) and ‘regulation of response to cytokine stimulus’ (GO:0060759).

## Discussion

Maternal recognition of pregnancy is an intriguing subject in the horse and extensive research on the molecular processes involved has been performed in the field of transcriptomics^7,18^. However, information on the downstream translation to proteins is scarce. In this study, quantitative proteomics of the uterine luminal fluid assessing the effect of pregnancy was performed for the first time in the horse. At the same time, proteins in the embryonic yolk sac fluid were mapped to provide insight into the embryo-maternal interaction.

**Figure 6:**
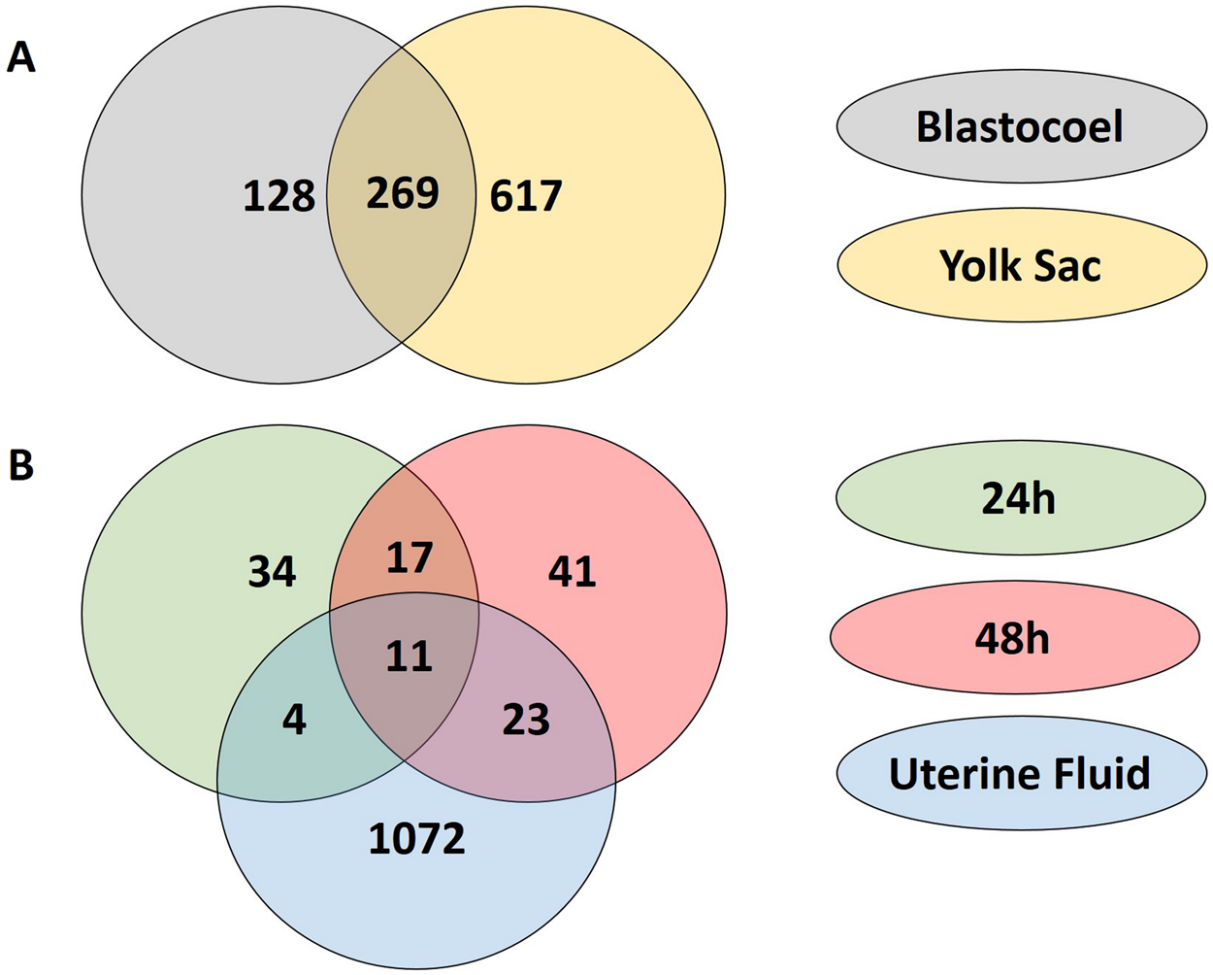
Comparison of proteins detected in the uterine fluid (UF) and the yolk sac (YS) with the proteins reported by Swegen *et al*.^34^. Figure 6A shows the number of proteins which were found in the blastocoel fluid by Swegen *et al*.^34^ and the YS in our study and Fig. 6B illustrates the proteins those to be secreted in embryo-conditioned medium at 24 h and 48 h by Swegen *et al*.^34^ and in the UF in our study. Numbers are based on the reported gene symbols.

With 119 proteins differentially expressed in the uterine fluid of P versus C mares, a distinct influence of pregnancy was established. In general, a function of more than 40% of the differentially expressed proteins in the UF was categorized as ‘binding (GO:0005488)’, coinciding with the findings in pigs and cattle, where the majority of proteins were also allocated to molecular binding^24,28^ (Fig. 1). ‘Binding’ also represents the main category to which the common proteins in UF and YS were allocated, with subtly higher representation of proteins upregulated during pregnancy in categories linked to embryo-maternal interaction, namely ‘developmental process (GO:0032502)’, ‘response to stimulus (GO:0050896)’, ‘transporter activity (GO:0005215)’ and ‘transcription factor activity - protein binding (GO:0000988)’ at the expense of the more general GO term ‘structural molecule activity (GO:0005198)’ (Fig. 3). Cellular component categorization allocated 45% of the identified UF proteins to the extracellular space (Fig. 2). This coincides with the findings of Swegen *et al*.^34^, who specifically targeted secreted proteins by analysing embryo-conditioned medium. This supports the fact the proteins detected in our study mainly represent the proteins secreted in the uterine fluid rather than endometrial cells shed in the uterine lumen. This also accounts for the proteins which were found to be differentially expressed during pregnancy. Sixty four % of these proteins were categorized in the extracellular space; the other may have originated from occasional shedding of embryonic and endometrial cells into the uterine lumen. Figure 2 represents an overview of all UF specific, YS specific and common proteins, including their differential expression in P vs C and their allocation to the extracellular space. Interestingly, the majority of proteins commonly found in UF and YS are indeed present in the extracellular space. These represent candidate proteins involved in embryo-maternal interaction and signalling. In general, our results greatly coincided with the findings of Swegen *et al*.^34^ who worked with day 8 blastocysts to examine proteins present in and secreted by early equine embryos. Figure 6 shows the number of proteins which were commonly found in the blastocoel fluid and the YS and those found to be secreted in embryo-conditioned medium at 24 h and 48 h and in the UF in our study. More than two third of the proteins reported in the blastocoel fluid were also detected in the YS and more than one third of the proteins found to be secreted after 48 h of embryo culture were also detected in the UF. Overlap of the results validates our independent findings on the one hand and indicates conserved expression of several proteins throughout development on the other hand.

**Figure 7:**
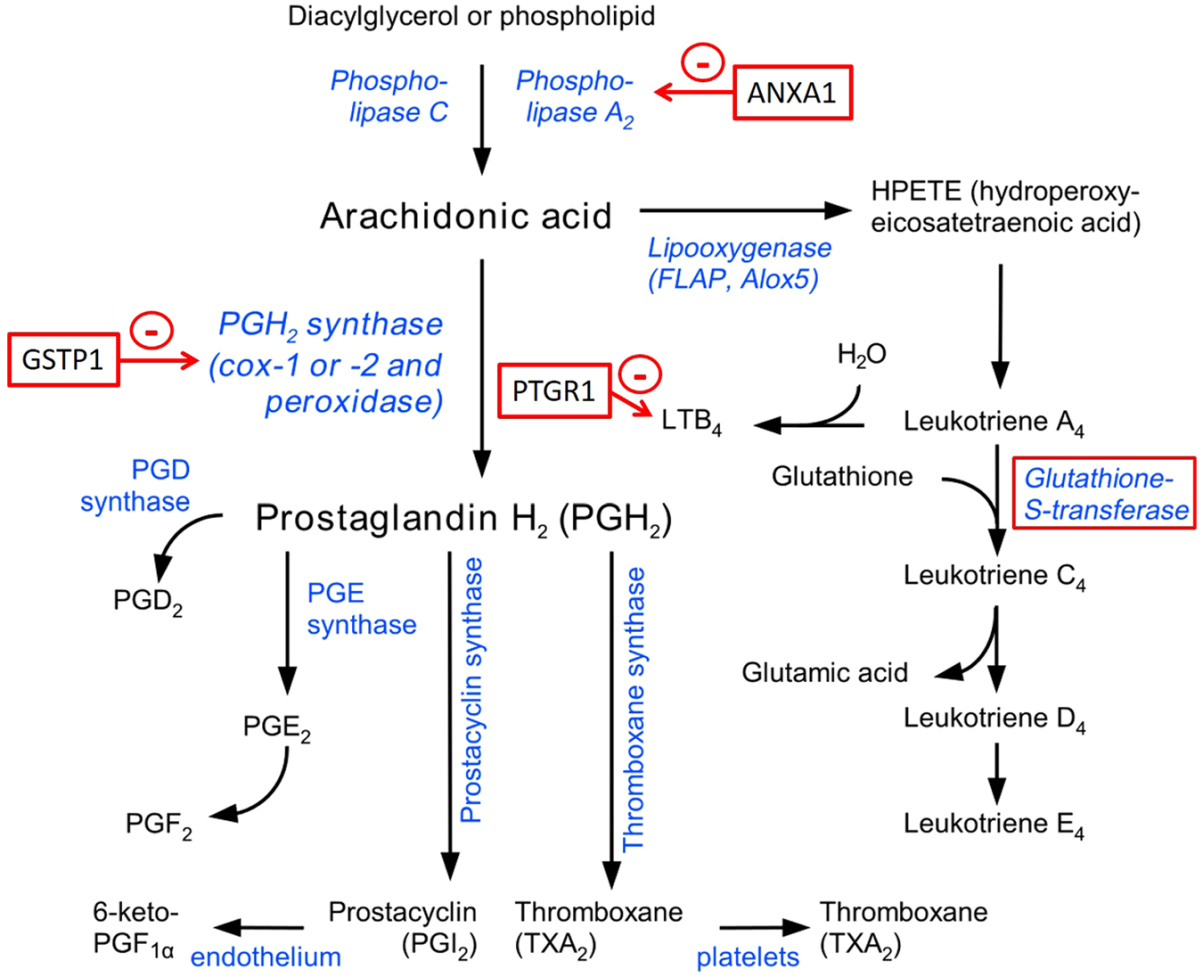
Inhibitors of prostaglandin synthesis in uterine fluid of pregnant mares. Eicosanoid pathway, adapted from Wikipedia. Proteins found to be upregulated in the uterine fluid of pregnant mares are marked in red.

In the context of MRP, prostaglandin synthesis is of special interest. For three proteins involved in this pathway, namely prostaglandin reductase 1 (PTGR1), glutathione transferase 1 (GSTP1) and annexin A1 (ANXA1), significantly higher amounts were detected in the uterine fluid of pregnant mares compared with cyclic mares. Apart from acting on 15-oxo-PGE1, 15-oxo-PGE2 and 15-oxo-PGE2-alpha as 15-oxo-prostaglandin 13-reductase, PTGR1 catalyzes leukotriene B4 into its biologically less active metabolite, being the key step in the metabolic inactivation of leukotriene B4, as depicted in Fig. 7.

While glutathione transferases are also generally involved in the biosynthesis of prostaglandins and leukotrienes, as well as progesterone and testosterone^36^, a specific anti-inflammatory effect of GSTP1 by reduction of PTGS2, formerly known as cyclooxygenase-2 (COX-2), has been described^37^. Furthermore, transport of GSTP1 across the plasma membrane was demonstrated^37^. Based upon these observations with recombinant human GSTP1 in mice and the high homology of equine GSTP1 with other species, equine GSTP1 in uterine fluid might cross the plasma membrane and target intracellular PTGS2. Interestingly, GSTP1 was also detected in the YS of the equine conceptuses. In this regard, the pregnancy associated upregulation of GSTP1 observed in the equine uterine fluid could be involved in the luteostatic mechanism by inhibiting PTGS2. Further research is needed to examine this hypothesis, as this is the first report on the presence of GSTP1 in equine uterine fluid.

Another anti-inflammatory factor with an inhibitory effect on prostaglandin synthesis, more specifically on phospholipase A2, is annexin A1 (ANXA1)^38,39^. Annexin A1 was upregulated in the uterine fluid of the pregnant mares when compared to the cyclic mares and this association of annexins with pregnancy coincides with literature. An increase in ANXA1 was also reported in the uterine luminal fluid of pregnant ewes from day 10 to day 12^25^. Several annexins have been linked to embryo-maternal interaction. Annexin 4 (ANXA4) was found to increase over time from day 10 to day 13 in both cyclic and pregnant pigs^24^, and we previously reported greater quantities of ANXA4 in the oviductal fluid of pregnant mares, when compared to cyclic mares^40^. In our study, we detected annexin 1, 2, 3, 4, 5, 7 8 and 11 in the UF, while ANXA2 and ANXA5 were also found in the YS. Swegen *et al*.^34^ also reported the presence of ANXA2 in both the equine blastocoel fluid and the embryo-conditioned medium after 48 h. The only annexin found to be upregulated during pregnancy was ANXA1. Annexin 1 is an inhibitor of phospholipase A2, a rate-limiting enzyme which liberates arachidonic acid for the synthesis of prostaglandins and for which a lower enzyme activity of phospholipase A2 has been demonstrated in pregnant mares compared to cyclic mares on day 14^41^. In our study, phospholipase A2 group IIA (PLA2G2A) tended to be downregulated in the uterine fluid of pregnant mares with a logFC of −1.31 compared to the cyclic condition, but it was not significant at a 0.05 FDR. Both PLA2G2A and phospholipase A2 group VII (PLA2G7) were detected in the YS; the latter was also found in the equine embryo-conditioned medium after 48 h^34^. Overall, our data suggest pregnancy associated interference with the luteolytic eicosanoid pathway with upregulation of inhibitory factors at different levels of the prostaglandin synthesis pathway.

A close interaction between prostaglandins and oxytocin has been described in the context of MRP in the horse with downregulation of the oxytocin receptor protein in the pregnant endometrium on day 14^13^. In the present study, the presence of oxytocin in the uterine luminal fluid was examined, but it was not detected. However, this does not mean it was not present in the original samples; the collection method might have retained some peptides and missing values are intrinsic to mass spectrometry^42,43^. Phosphoinositide phospholipase C (PLCD1), involved in the oxytocin receptor signalling pathway, was detected, but not significantly affected by pregnancy^44^. The reduced expression of *OXTR* during pregnancy has been hypothesized to be induced by an observed decrease in the gene expression of oestrogen receptor 1 (*ESR1*) in the pregnant equine endometrium^15^. Several proteins related to ESR1 were also found to be affected in the uterine fluid. Surprisingly, pregnant mares showed a strong upregulation of deoxynucleotidyltransferase terminal interacting protein 2 (DNTTIP2), previously known as oestrogen receptor binding protein (ERBP). Binding of DNTTIP2 to ESR1 enhances its transcription^45^. Upregulation of DNTTIP2, which would lead to increased transcription of *ESR1* in pregnant mares is contradictory to findings in literature and further targeted research is required to clarify this aspect. Downstream of the ESR1, the influence of oestrogen on the ezrin–radixin–moesin (ERM) family of actin-binding proteins has been studied, mainly in the context of breast cancer^46,47^. The distribution pattern of ERM-proteins in the blastocyst and the uterus has been linked to the implantation potential in mice. Protein analysis of uterine fluid has demonstrated the presence of ezrin (EZR) and moesin (MSN) in cattle^26,27^ and pigs^23^. In the horse, upregulation of EZR and downregulation of MSN was detected, while an inverse association with pregnancy was noted in cattle^26,27^. We detected both EZR and MSN in the YS and they were also found in the blastocoel fluid^34^.

Apart from the specific interest in proteins involved in prostaglandin synthesis, we also aimed to create a general overview of the proteins present at the embryo-maternal interface and potentially involved in signalling. Supplementary File 4 presents all proteins which were commonly found in the UF of P mares and in the YS and the functions of each protein are included. To visualize their role in embryo-maternal interaction and signalling, the proteins involved in GO terms including ‘embryo’, ‘maternal’ or ‘uterus’ are depicted in Fig. 4 and those linked to GO terms ‘growth factor’ and ‘cytokine’ in Fig. 5. The origin of the proteins can be distinguished in red (UF), yellow (YS) and pink (UF and YS) and up- and downregulation during pregnancy is represented by enlargement or shrinkage of the protein respectively. Interestingly, most proteins which were found to be upregulated during pregnancy were detected both in UF and in YS, while downregulation during pregnancy generally coincided with absence of these proteins in the YS, indicating a potentially important role of the embryo in the production of these proteins during pregnancy. Several common proteins were found at the embryo-maternal interface during MRP in cattle, including aconitase 1 (ACO1), which was specifically detected in the uterine fluid of pregnant and not in cyclic heifers, as well as glucose-6-phospate isomerase (GPI), which has been detected in the uterine fluid of both pregnant and cyclic heifers and for which an embryonic source has been presumed based on transcriptomics^27^. In our study, both proteins were found in the YS and the UF of P mares, with significant upregulation of GPI in P versus C. Two other proteins which were commonly found in UF and YS, namely FK506 binding protein 4 (FKBP4) (Fig. 4) and heat shock protein 90 (HSP90AB1) (Fig. 5), have been elaborately discussed by Swegen *et al*.^34^ concerning their progesterone supportive role. Co-operation of both factors is necessary for activation of the progesterone receptor^48^, FKBP4 has shown to be crucial for uterine receptivity and implantation in mice^49^ and FKBP4 deficit has been associated with pregnancy loss in human^50^. While FKBP4 was detected in equine blastocoel fluid and speculated to be involved in signalling, it was not detected in the embryo-conditioned medium^34^. Interestingly, we did find both FKBP4 and HSP90AB1, not only in YS, but also in UF, even though their presence was not affected by pregnancy. While further confirmation of the role of specific proteins is required, the overview created in this study can be used as a basis for further targeted studies in the horse.

In addition to the role in prostaglandin and progesterone metabolism, involvement in proteolysis and lipid metabolism was also prominent in the commonly detected proteins in our study and the one of Swegen *et al*.^34^, also coinciding with previous findings on transcriptomics around MRP^15^. Several cathepsins (G, D, L and S) were detected in the UF with downregulation of cathepsin L (CTSL) and S during pregnancy. Pregnancy associated downregulation of *CTSL1* was also found at the transcriptome level^15^. Considering lipid metabolism, we detected differential expression of lipocalin (P19), apolipoprotein A1 (APOA1) and apolipoprotein D (APOD). These proteins are important transporters of essential lipids to the developing conceptus. Retinol binding protein (RBP), which also belongs to the lipocalin family, and APOA1 have been detected in uterine fluid of pregnant and cyclic pigs, cattle and sheep^23–25,27,28^, with increasing amounts between day 10 and day 13 in both pregnant and cyclic pigs^24^. In the horse, lipocalin (P19), apolipoprotein A1 (APOA1) and apolipoprotein D (APOD) were all downregulated in the uterine fluid of the pregnant mares. Pregnancy associated upregulation of *APOA1* was reported at the transcriptome level^15^ and presence of P19 and APOA1 in the yolk sac fluid illustrates their role in the embryo-maternal dialogue. Therefore, lower amounts in the uterine fluid during pregnancy rather indicate the transport and binding to the conceptus. Lipocalin P19 or uterocalin is a progesterone induced protein, which is abundantly present in the equine uterine secretions during dioestrus and early pregnancy^51,52^. While the early developing equine conceptus moves around the uterus, it entirely depends upon the uterine secretions for its nutrition and P19 can function as a carrier for essential lipids and amino acids^53^. Coinciding with our findings, P19 has been detected in the trophoblast and the yolk sac fluid of the equine embryo^51,52^ and it is one of the most abundant proteins in the embryonic capsule^54–56^. Therefore, the lower amount of P19 in P versus C is probably due to binding of substantial quantities to the embryo.

While a novel and informative overview is created, it has to be borne in mind that no statistically significant results were obtained at the level of molecular functions, biological processes and pathways. Differential expression of individual proteins was observed between the different UF conditions, and these proteins were categorized in GO terms, but statistical analysis showed no significant overrepresentation of any of the GO terms or KEGG pathways. Furthermore, it should be noted that MS intrinsically suffers from missing values and conclusions based on the absence of proteins cannot be made^42,43^. However, the field of proteomics has greatly evolved in recent years, providing the possibility for statistically robust quantitative comparison of individual protein levels in complex biological samples, like uterine fluid^22^. HDMSE specifically has been shown to provide good proteome coverage and reproducibility^35^. At the same time, however, analysis of GO terms and pathways for proteomics is still in its infancy^57,58^. As many of the here described bioinformatics approaches for proteomic analysis were originally developed for genomics, a similar but more matured field, their performance can be expected to show a similar growth as that of the genomic approaches. Moreover, the similarity between these fields potentially allows an integrated approach in which results from several omics studies can be combined.

In conclusion, proteins present in the equine uterine and embryonic yolk sac fluid around the signalling of MRP at day 13 were identified and quantified at large scale for the first time in the horse. We detected upregulation of several inhibitors of prostaglandin synthesis, including PTGR1, GSTP1 and ANXA1, in the uterine fluid of pregnant mares. Overall, an overview was created of the proteins playing a role at the embryo-maternal interface in the horse. This study provides a solid foundation for further targeted studies of proteins potentially involved in embryo-maternal interactions, maternal recognition of pregnancy and pregnancy loss in the horse.

## Methods

### Sampling

All animal handlings were approved by the Ethical Committee of the Faculty of Veterinary Medicine (EC2013/118) of Ghent University. All methods were performed in accordance with the relevant guidelines and regulations. A switch back design was followed with 5 mares undergoing two different types of cycles: a pregnant cycle (P) and a cyclic control cycle (C). In this way, the samples were paired using the same mare as its own control for pregnancy and the experimental unit was the mare. The order of P and C cycles was randomly altered for the different mares. No resting cycles were included. During the breeding season, five reproductively sound Warmblood mares between 4 and 13 years old were monitored by transrectal ultrasound. Reproductive soundness was confirmed by negative cytology and bacteriology. Mares displaying uterine oedema and a follicle exceeding 35 mm received 1500 IU hCG intravenously and were either inseminated the next day with fresh semen of the same stallion (P) or left unbred (C). Ovulation was evaluated twice daily by ultrasound. In both groups, sampling was performed 13 days after detection of ovulation. To recover undiluted uterine fluid in order to avoid negative effects of excessive Ringer’s salts on MS^59^, intra-uterine application of a tampon (OB Mini; Johnson & Johnson, Beerse, Belgium) was performed based upon the method described by Wolf *et al*.^33^. A double gloved technique was used to avoid vaginal contamination. The tampon was left in the uterus during 10 minutes and upon removal it was placed in a Falcon tube at 4 °C until further processing. Subsequently, the mare’s uterus was flushed with sterile Ringer’s solution by means of a modified endotracheal tube to recover the embryo (P).

To process the uterine fluid, 1 mL of sterile water (B60, Biosolve, Valkenswaard, The Netherlands) was infused on top of the tampon and the tampon was attached in the upper part of the Falcon tube by fixing the cord with the cap. Subsequently, the Falcon tube was centrifuged for 20 minutes at 1000 × g at 4 °C. The supernatant was collected and stored in a Protein LoBind Eppendorf tube (Eppendorf AG, Hamburg, Germany) at −80 °C. Meanwhile, the embryo was isolated in a petri dish and the yolk sac fluid was collected by aspiration with a 21 G needle and stored in a Protein LoBind Eppendorf at −80 °C.

A total of 15 samples were collected, consisting of uterine fluid (UF) (n = 10) from five biological replicates coinciding with the five mares (1–5) for the P and C treatment cycles, as well as yolk sac fluid (YS) (n = 5) from the P cycles.

### Sample preparation for mass spectrometry analysis

After thawing, protein concentration in each sample was determined using the Coomassie (Bradford) Protein Assay Kit (Thermo Fisher Scientific, San José, CA, USA) according to the manufacturer’s instructions. Further processing was performed for 10 µg protein of each uterine fluid sample and for 500 ng protein of the yolk sac samples. Samples were dissolved in 20 μL 0.5 M triethylammonium bicarbonate (TEABC; Sigma-Aldrich, St. Louis, MO, USA). Two µl of reducing agent (10 µM DTT; Invitrogen, Merelbeke, Belgium) were added followed by incubation for 1 h at 60 °C. Subsequently, 1 µl of alkylizing agent (200 mM methyl methanethiosulfonate (MMTS) in isopropanol; Sigma-Aldrich, St. Louis, MO, USA) was added and samples were incubated for 10 min at room temperature. Digestion was performed overnight at 37 °C with trypsin lys C (1:20, trypsin:protein w/w, Promega, Leiden, The Netherlands) in TEABC buffer with 1 mM CACL(2) and 5% acetonitrile (Biosolve, Valkenswaard, The Netherlands). Samples were vacuum-dried and stored at −20 °C until analysis.

### Data acquisition by HDMS^E^ analysis

The peptides were separated using a nanoscale UPLC system (nanoAcquityUPLC, Waters, Milford, USA) coupled to a Synapt G2-Si mass spectrometer (Waters). Peptides were first trapped in 0.1% formic acid on a 180 µm × 20 mm C18 Trap column. Separation was performed on a HSS C18 1.8 m, 100 m × 250 mm analytical column at a flow rate of 300 nL/min and a temperature of 45 °C. As mobile phase A a 0.1% formic acid with 4% DMSO in water solution was used and 80% ACN containing 0.1% formic acid constituted mobile phase B. Peptides were separated for 60 min at 1–40% solvent B and for 1 min 40–85% solvent B. Seven minutes of rinsing (85% solvent B) re-equilibrated the column to the initial conditions. Eluted peptides were analysed in positive mode ESI-MS using High Definition MS^E^ (HDMS^E^) with a collision energy look up table as described in^22^. The spectral acquisition time of low and elevated energy scans was 0.6 s over an m/z range of 50–2000. [Glu1]-Fibrinopeptide B was used for post-acquisition lock mass correction. All UF samples were analysed in the same run; three technical replicates (R1–R3) were run for each sample and four quality controls (QC) were included in which all samples were pooled.

### Identification and quantification of peptides and proteins

All data were processed in Progenesis QIP (Progenesis QIP 2.0, Nonlinear Dynamics, Waters), including normalization and quality control. A database with UniProt IDs was created by conversion of Ensembl gene identifiers for Equus caballus (n = 22295) to Uniprot IDs using http://www.uniprot.org/uploadlists/ and including common contaminants (http://www.thegpm.org/crap/). As only secreted proteins are expected to be found, it can be argued that this database should be limited to only these secreted proteins. However, there is much debate on the accuracy of FDR calculations with such limited databases^60–62^ and as such a cautious approach was taken in which all proteins were assessed. Using Progenesis QIP, peptides were identified against this database with a FDR of 4%^63^ and allowing maximum one miscleavage. Protein quantification was based on the Hi-3 method^64^, which uses the average of the three most intense peptides of each protein for its quantification. Resulting normalized abundances for each protein, as well as unique peptide counts were further used for analysis of differential expression.

### Analysis of differential expression

Analysis of differential expression was performed for the UF samples. Only normalized abundancies of proteins with at least two unique peptides (n = 707) were included in the analysis. Pairwise comparisons of differential expression were made for P versus C with the individual horses as a blocking factor, using R Bioconductor limma package^65^ and a FDR of 0.05.

### Gene Ontology enrichment and pathway analysis

Gene Ontology (GO) terms (molecular functions, biological processes and cellular locations) were downloaded for each protein with the PANTHER Classification System^66^. For the pair-wise comparison of P and C, a statistical overrepresentation test against all quantified proteins was performed for all significantly up- and downregulated (FDR < 0.05) proteins. These tests were done for all primary GO classes: molecular functions, biological processes and cellular components. Pathways were analysed with Bioconductor’s^67^ GAGE package^68^. LogFC values of all quantified proteins were used as input against Equus caballus background reference pathways from KEGG.

Proteins involved in the embryo-maternal interaction were visualized using Cytoscape 3.3.0. To visualize embryo-maternal signalling, all GO terms and pathways that include ‘cytokine’ or ‘growth factor’ in their description were selected, together with all identified proteins in either the yolk sac or uterine fluid belonging to these GO terms or pathways. The connection between these GO terms, pathways and proteins was then visualized using Cytoscape 3.3.0. The same methodology was used to create a network based on GO terms and pathways including ‘embryo’, ‘maternal’ or ‘uterus’ in their description.

### Data availability

All data are available in the Supplementary files.

## Electronic supplementary material

Supplementary file 1, 2, 3, 4
